# Liquid-Assisted Femtosecond Laser Precision-Machining of Silica

**DOI:** 10.3390/nano8050287

**Published:** 2018-04-28

**Authors:** Xiao-Wen Cao, Qi-Dai Chen, Hua Fan, Lei Zhang, Saulius Juodkazis, Hong-Bo Sun

**Affiliations:** 1 State Key Laboratory of Integrated Optoelectronics, School of Mechanical Science and Engineering, Nanling Campus, Jilin University, Changchun 130025, China; xw2015@outlook.com (X.-W.C); zhanglei@jlu.edu.cn (L.Z.); 2 State Key Laboratory of Integrated Optoelectronics, College of Electronic Science and Engineering, Jilin University, Changchun 130012, China; chenqd@jlu.edu.cn (Q.-D.C.); fanhua17@mails.jlu.edu.cn (H.F.); 3 Centre for Micro-Photonics, Faculty of Science, Engineering and Technology, Swinburne University of Technology, Hawthorn, VIC 3122, Australia; sjuodkazis@swin.edu.au; 4 Melbourne Centre for Nanofabrication, ANFF, 151 Wellington Road, Clayton, VIC 3168, Australia; 5 State Key Laboratory of Precision Measurement Technology and Instruments, Department of Precision Instrument, Tsinghua University, Haidian, Beijing 100084, China

**Keywords:** femtosecond laser, silica, Laser materials processing, nonlinear optics at surfaces

## Abstract

We report a systematical study on the liquid assisted femtosecond laser machining of quartz plate in water and under different etching solutions. The ablation features in liquid showed a better structuring quality and improved resolution with 1/3~1/2 smaller features as compared with those made in air. It has been demonstrated that laser induced periodic structures are present to a lesser extent when laser processed in water solutions. The redistribution of oxygen revealed a strong surface modification, which is related to the etching selectivity of laser irradiated regions. Laser ablation in KOH and HF solution showed very different morphology, which relates to the evolution of laser induced plasma on the formation of micro/nano-features in liquid. This work extends laser precision fabrication of hard materials. The mechanism of strong absorption in the regions with permittivity (epsilon) near zero is discussed.

## 1. Introduction

Femtosecond (fs) laser has proved to be an efficient tool for micro/nanomachining [1] in micro-optics [2], micromechanics [3], microfluidics [4], organic light-emitting diode (OLED) display [5], and micro-sensing [6]. Based on the nonlinear nature of light–matter interaction via multi-photon and avalanche absorption [4,7], fs-laser machining is independent of the material’s hardness and has been demonstrated on a wide range of metals, semiconductors, and dielectrics [8,9,10]. By intense laser pulses focused with an objective lens, structures and patterns of 2D and 3D morphology have been realized [2,8]. However, it also has disadvantages. The laser–matter interaction at the surface is inevitably affected by the evolving ablation pattern of the fabricated structure due to the scattering and absorption, also chemical modification [11]. The laser-induced ripples generated by an imprint of a plasmonic wave [12,13] would greatly increase the surface roughness which can be desired depending on application. Also, laser ablated debris randomly falling on the surface could enhance the light absorption and scattering [9,14]. Especially when laser induced thermal effects are pronounced [15,16], debris is difficult to wash out after laser fabrication. To solve these problems, fs-laser-assisted etching method [17,18] and liquid-assisted fs-laser machining [19,20] have been proposed as two possible solutions. The former method uses laser-induced selective etching to remove the modification area without leaving debris. While the latter uses the laser induced shock wave and water plasma generating cavitation bubbles to wash the debris. When a laser beam is focused on the sample surface through the liquid, subsequent laser pulses are scattered by the bubbles which is a disadvantage for precision machining [10]. For geometry when light is focused through a transparent substrate onto an interface with liquid, light-induced backside wet etching (LIBWE) is realized as originally developed for optical projection processing [21,22]. We adopted LIBWE for the direct laser writing. LIBWE delivers a better surface morphology of fs-laser processed areas in liquid. However, the surface quality (smoothness) after laser-assisted etching would suffer from loss in resolution and departure from the initial design due to chemical enhancement of material removal. Although debris can be removed in the liquid-assisted fs-laser machining, surface roughness is affected by the periodic structures [23].

In this paper, we report a systematical study of liquid assisted fs-laser machining of quartz plates in water, KOH, and HF solutions as explored in LIBWE geometry. The obtained micro-holes in liquid have a better morphology and a smaller diameter (1/3~1/2) compared with those fabricated in air. It has been found that the formation of laser induced periodic structures was decreased in solutions. Due to the redistribution of oxygen at the laser ablation site, chemical reactions affected formation of the induced periodic structures and provided better control over surface modification.

## 2. Materials and Methods

**Figure 1:**
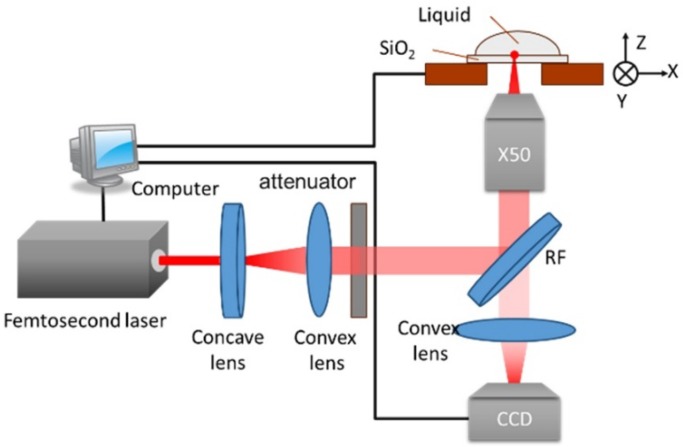
Schematics of the liquid-assisted femtosecond laser precision-machining system. The 50× magnification objective lens was uses, RF is the reflector. A CCD (charge-coupled device) was used for real-time observation of laser processing.

The schematics of the employed experimental laser microfabrication system are shown in Figure 1. A regeneratively amplified Ti:sapphire laser was used, which delivered pulses with a duration of 100 fs (FWHM), center wavelength of 800 nm, and repetition rate of 500 Hz. The diameter of the beam was approximately 6 mm, and then expanded to 18 mm with a beam expander, which was composed of two lenses with focal lengths of −50 mm and 150 mm. The laser beam was focused onto the upper surface (in contact with solution) through the sample using an objective lens (50× magnification, numerical aperture NA of 0.7 and aperture of 9 mm). The pulse energy was measured at the exit pupil of the objective lens. A quartz plate, 1-mm-thick, was mounted on a 3D translation stage with a positioning resolution of 0.1 μm (Figure 1). The upper surface was in contact with various solutions: deionized water, KOH (5% and 40% by mass ratio), and HF solution (2% or 5 mol/L). In this LIBWE case, the focused spot would not be influenced by bubble formation during laser fabrication. The laser fluence of the incident pulses was continuously tuned using a variable attenuator, while the laser pulse number was controlled by a shutter with a temporal resolution of 1 ms, which made it possible to obtain a single pulse.

After laser fabrication, the sample was cleaned in an ultrasonic bath with KOH solution (40%, mass concentration) at room temperature, which removed all the debris from the surface thoroughly. After cleaning, the morphology of the ablated region was obtained with a scanning electron microscope (SEM, JSM-7500F, JEOL Ltd., Akishima-shi, Japan). At each machining parameter, a 5 × 5 hole-array was fabricated and all the results were the average of these 25 dots.

## 3. Results and Discussion

**Figure 2:**
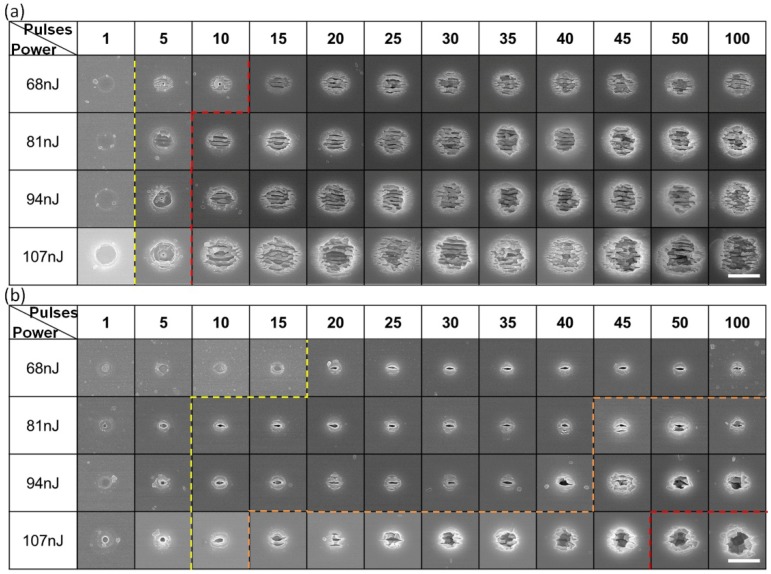
Scanning electron microscope (SEM) images of holes induced in air (**a**) and water (**b**) by different number of pulses varied from 1 to 100 and pulse energy ranging from 68 nJ to 107 nJ. Scale bars are 2 μm.

At first, the morphology of the holes fabricated with different laser pulse energies and pulse numbers were investigated after fabrication in air and deionized water. As shown in Figure 2, the laser pulse energy was set from 68 nJ to 107 nJ and the pulse number was changing from *N* = 1 to 100. Apparently, it indicates that the size of the holes ablated in deionized water was much smaller than those produced in air ambient (in LIBWE geometry). The interior of holes made in deionized water were relatively cleaner. In air, an unstructured ablation hole was observed at one pulse regardless of the pulse energy. Grating-like ripples with an orientation perpendicular to the laser polarization (the normal ripples) [24] were found around the irradiated regions at exposures above five pulses and 68 nJ/pulse energy. It is worth noting that a smaller ablation hole with diameter of about 120 nm was observed in the center of the irradiation region at 5–10 pulses of 68 nJ. There was still a pronounced ablation hole at the center for exposure by five pulses of 94 nJ and 107 nJ, which may be due to the competition between laser ablation and ripple formation as discussed later in more detail. When the pulse number was more than 10, laser induced ripples dominated the morphology regardless of the laser pulse energy.

In water, the ablated products may interact and undergo chemical modifications in a short time after the laser pulse, especially inside the cavitation bubble [25]. Compared with air, the atom density of water is three orders of magnitude higher and confines the laser shock energy and plasma plume to a smaller region. As aforementioned (Figure 2a), ablation holes were observed with a smaller diameter after the first pulse regardless of the pulse energy. However, the hole formation by the ablation continued till 100 pulses at 68 nJ, 40 pulses at 81 nJ, 40 pulses at 94 nJ, and 10 pulses at 107 nJ and dominated laser structuring and material removal. Competition between direct hole formation by laser ablation and ripple formation was refined on larger areas and larger numbers of laser pulses. Ripples were printed directly on the surface as the ablation hole changed from circular, to elliptical, to linear and, finally, along the linear central feature (Figure 2b). This revealed a systematic change of surface modifications when all focusing conditions are same. 

**Figure 3:**
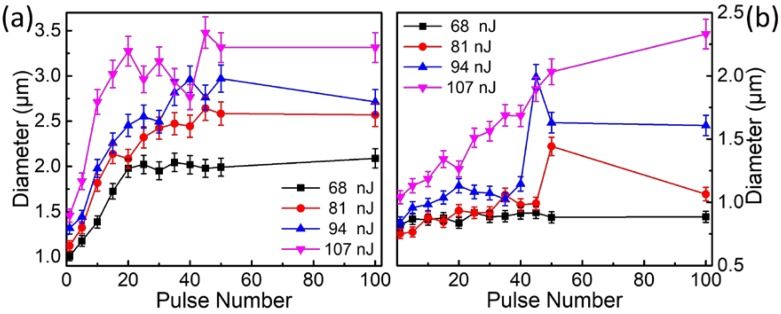
Diameter of the ablated area with pulse number in air (**a**) and water (**b**). Diameter is defined as a recognizable surface damage cross section in SEM images.

The diameter of the ablated area with the pulse number in air (a) and in water (b) is shown in Figure 3. In air, the diameter increased quickly with the pulse number below 30 pulses and then maintained saturation until 100 pulses. In water, the diameter changed much slower as the pulse increased at energies of 68 nJ, 81 nJ, and 94 nJ. However, it went up linearly with pulse accumulation at 107 nJ, which could also be observed in Figure 2b. The ablation threshold fluences of the quartz plate in air and water were evaluated by investigating the dependency of the ablated size (squared diameter, *D*^2^) on the irradiation pulse energy. By fitting the data according to the equation (assuming Gaussian intensity profile) [26]

(1) $$D^{2}=2\omega_{0}^{2}\ln(E_{p}/E_{th})$$

where *ω*_0_ is the beam waist (radius) at the focal plane and *E_th_* is the threshold energy. The ln(*E_th_*) could be obtained as asymptotic value when *D*^2^→0. The threshold fluences, *ϕ_th_*, could be calculated for the peak amplitude

(2) $$\phi_{th}=2E_{th}/\pi \omega_{0}^{2}$$

For a single shot, the calculated threshold pulse energies were about 46.4 nJ and 35.7 nJ in air and in water, respectively, which corresponded to the fluences of 2.3 J/cm^2^ and 5.3 J/cm^2^, respectively.

**Figure 4:**
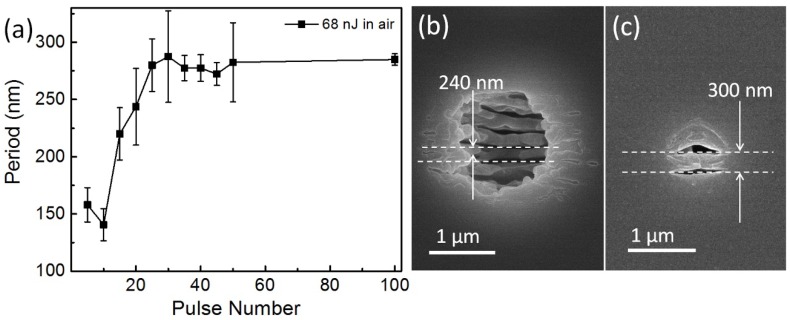
Laser induced periodic structures in air. (**a**) The period evolution at 68 nJ; (**b**) period at 50 pulses and 81 nJ in air; (**c**) period at 50 pulses and 81 nJ in water.

Laser induced periodic structures were found depressed in water as shown in Figure 2 with more details in Figure 4. Taking the period at 68 nJ, for example, the period observed at the ablation center at 5 or 10 pulses is around 150 nm. It quickly increased to around 260 nm at 30 pulses. Then it kept the saturation value up to 100 pulses. If the pulse energy increased, as shown in the Figure 4b, the center period at 50 pulses was around 289 nm at 81 nJ. While as a case in water, the period at 45 pulses and 68 nJ was around a slightly larger period of 300 nm. This is attributed to the stronger ablation which induced structures deep into the sub-surface volume. The induced nanostructures were formed by the accepted model of a standing wave at the interface of the active plasma (excited) layer and silica host (the refractive index around 1.48) rather than at the silica–air interface. The ambient refractive index has been demonstrated to be an important factor to the period of the induced structures; the smaller the refractive index, the larger the period [27,28].

From the cross-section, it can be seen that the depth of periodic structures extended a micrometer into silica. Interestingly, the structures become squeezed with smaller periods at larger depths, expandable by the effective medium theory, i.e., a larger effective refractive index more far from the ablated interface. 

**Figure 5:**
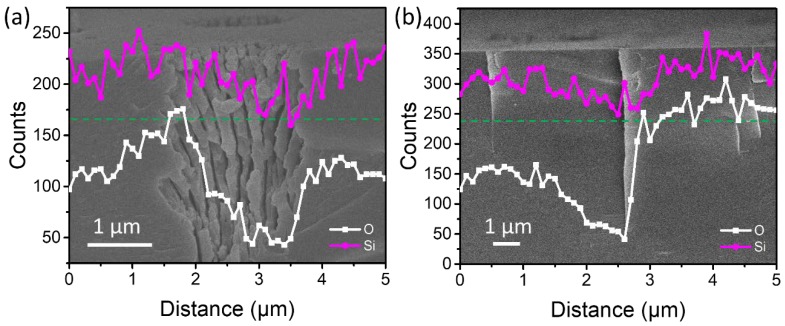
O and Si content along the depth of ablated surface (**a**) in air and (**b**) in water. The background SEM image has the same axial and lateral scales as the compositional plot.

The redistribution of the oxygen revealed lower concentration in the center but higher at the ablation border by energy disperse spectrum (EDS) measurement, as shown in Figure 5a. In the water, the morphology in cross section was different. The ablation hole without nanostructures extended deep into the bulk. However, the oxygen presented a similar distribution as that in the air ablated samples (Figure 5b). Ablation of silica evolves via building up of the electrostatic field between the fast removed electrons from the surface and lagging ions. High temperature and pressure conditions are created [29], which would cause phase changes and ion separation during the stage of hydrodynamic movement after an ultra-short laser pulse.

**Figure 6:**
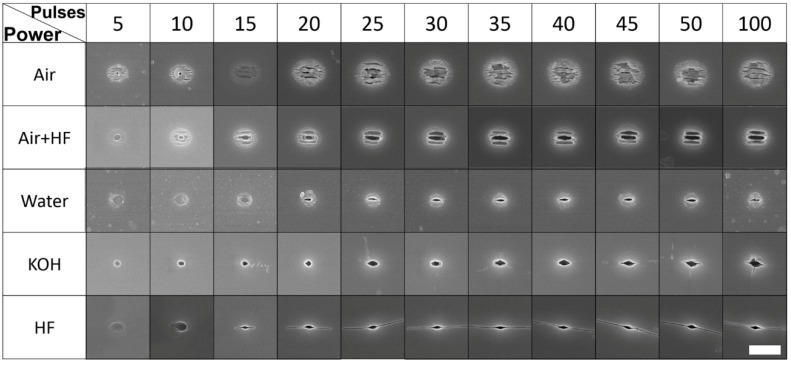
Comparison between ablations in different solutions. The pulse energy for each is 68 nJ and the scale bar denotes 2 μm.

The laser ablation process caused elemental redistribution, which is the most probable reason for selective etching of the ablated area and non-ablated area [30]. For example, the formed nanogratings in air would become different with larger nanovoids but could be made smooth after HF etching for 10 min, as shown in the second line in Figure 6. Slow etching at room temperature becomes accelerated by the ablation which is known also in LIBWE to cause cavitation (hence surface disintegration under negative tensile pressure). The ablation holes become rounder and deeper at the same pulse energies as compared with those in water (lines one and two in Figure 6).

The morphologies of laser ablation in the etching solutions were very different for the HF and KOH: the surface becomes cleaner without laser induced ripples. Nanovoids of elliptical shape similar with those water but with a nanogaps extending perpendicular with the laser polarization were observed for KOH and HF. This can be attributed to the electronic heat conductivity enhancement along the electrical component of the oscillating light E-field which facilitates chemical reaction and thermal diffusion [31]. E-field control of electronic transport during laser irradiation (rather diffusional all directional spreading) is expected to manifest itself as enhanced chemical etching of material deposition and can be linked to the convectional mechanisms in liquid environment [32].

Femtosecond laser machining is discussed as ‘cold processing’ [33] due to having a smaller heat affected zone. However, this is achieved due to well-controlled energy deposition and high intensity with temperatures reaching more than 500 °C [29] at the irradiated area, especially in the cavitation and bubble formation mode. High temperature enhances chemical reactions such as etching in KOH and HF solutions via the standard Arrhenius activation mechanism. The reaction in HF acid is more effective in material removal as compared with KOH since reaction product K_2_SiO_3_ is less dissolvable compared to SiF_4_.

A unique feature of the laser ablated patterns is the well-centered hole formation especially recognizable with smaller numbers (*N* = 5) of pulses (see Figure 2 and Figure 6). The pronounced central deeper ablation holes can be explained by the energy deposition. The strongest light absorption takes place at the regions where permittivity (epsilon) is near zero (ENZ) [34]. This occurs at the very central part and can explain the formation of stronger ablation which is localized into the sub-diffraction area. Since ENZ regions depend on the permittivities of the host as well as the free electron plasma, this feature of strong localization of ablation can be engineered and will be the focus of future studies.

## 4. Conclusions

In summary, we have systematically investigated the interaction of fs-laser pulses with fused quartz (silica) in air, water, KOH, and HF solutions in LIBWE geometry. Smaller holes and smooth surface were obtained in water and water solutions. The threshold in water had a similar decreasing trend with pulse accumulation. In addition, laser induced periodic structures were less pronounced in water. Etching enhancement along the E-field of a linearly polarized laser beam was observed in the laser ablation in solution, which is attributed to the thermal effect enhanced chemical reaction and E-field enhanced electronic conductivity. Our systematical investigation opens up prospects for a better controlled high precision nano-/micro-scale fabrication of hard and chemically inert materials. The mechanism of energy deposition into the ENZ regions is discussed.
